# Applications of Fruit Polyphenols and Their Functionalized Nanoparticles Against Foodborne Bacteria: A Mini Review

**DOI:** 10.3390/molecules26113447

**Published:** 2021-06-06

**Authors:** Harsh Kumar, Kanchan Bhardwaj, Natália Cruz-Martins, Eugenie Nepovimova, Patrik Oleksak, Daljeet Singh Dhanjal, Sonali Bhardwaj, Reena Singh, Chirag Chopra, Rachna Verma, Prem Parkash Chauhan, Dinesh Kumar, Kamil Kuča

**Affiliations:** 1 School of Bioengineering & Food Technology, Shoolini University of Biotechnology and Management Sciences, Solan 173229, India; microharshs@gmail.com; 2 School of Biological and Environmental Sciences, Shoolini University of Biotechnology and Management Sciences, Solan 173229, India; kanchankannu1992@gmail.com (K.B.); rachnaverma@shooliniuniversity.com (R.V.); 3 Faculty of Medicine, University of Porto, Alameda Prof. Hernani Monteiro, 4200-319 Porto, Portugal; ncmartins@med.up.pt; 4 Institute for Research and Innovation in Health (i3S), University of Porto, 4200-135 Porto, Portugal; 5 Laboratory of Neuropsychophysiology, Faculty of Psychology and Education Sciences, University of Porto, 4200-135 Porto, Portugal; 6 Department of Chemistry, Faculty of Science, University of Hradec Kralove, 50003 Hradec Kralove, Czech Republic; eugenie.nepovimova@uhk.cz (E.N.); patrik.oleksak@uhk.cz (P.O.); 7 School of Bioengineering and Biosciences, Lovely Professional University, Phagwara, Punjab 144411, India; daljeetdhanjal92@gmail.com (D.S.D.); sonali.bhardwaj1414@gmail.com (S.B.); reena.19408@lpu.co.in (R.S.); chirag.18298@lpu.co.in (C.C.); 8 Lal Bahadur Shashtri, Government Degree College, Saraswati Nagar, Shimla 171206, India; ppchauhan321@gmail.com; 9 Biomedical Research Center, University Hospital Hradec Kralove, 50003 Hradec Kralove, Czech Republic

**Keywords:** fruit types, extraction of polyphenols, antibacterial activity, safety

## Abstract

The ingestion of contaminated water and food is known to cause food illness. Moreover, on assessing the patients suffering from foodborne disease has revealed the role of microbes in such diseases. Concerning which different methods have been developed for protecting food from microbes, the treatment of food with chemicals has been reported to exhibit an unwanted organoleptic effect while also affecting the nutritional value of food. Owing to these challenges, the demand for natural food preservatives has substantially increased. Therefore, the interest of researchers and food industries has shifted towards fruit polyphenols as potent inhibitors of foodborne bacteria. Recently, numerous fruit polyphenols have been acclaimed for their ability to avert toxin production and biofilm formation. Furthermore, various studies have recommended using fruit polyphenols solely or in combination with chemical disinfectants and food preservatives. Currently, different nanoparticles have been synthesized using fruit polyphenols to curb the growth of pathogenic microbes. Hence, this review intends to summarize the current knowledge about fruit polyphenols as antibacterial agents against foodborne pathogens. Additionally, the application of different fruit extracts in synthesizing functionalized nanoparticles has also been discussed.

## 1. Introduction

Food illness often occurs following the ingestion of contaminated water, food, chemicals, toxins, and pathogenic microbes (such asbacteria, viruses, parasites, and fungi) [1]. As per published literature, most foodborne illnesses are linked to bacteria (66%), and then by chemicals (26%), parasites (4%), and viruses (4%). The two highly common categories of foodborne illness are infections and intoxications [1].

Todate, around 200 diverse foodborne illnesses have been identified [2]. Predominantly, the severe cases of food illness have been observed in older people and infants as they do not have a robust immune system function, while in healthy individuals, high immune response has been observed following the intake of a high dosage of toxins and pathogens [2]. *Salmonella* and Campylobacter spp., are the main pathogens, and have been foundto be involved in >90% cases of food illness, thus being proclaimed to be responsible for food hazards globally [3]. Table 1 lists the outbreaks associated with foodborne bacteria.

Numerous preservation techniques have been developed to maintain the food safety from microbes, the sensory characteristics, and the nutritional value of food. In few cases, these techniques exhibit undesired effects in both organoleptic and nutritional features; moreover, synthetic preservatives have also been associated with allergic reactions, with benzoates, formaldehyde, nitrates, phenolic antioxidants, sorbates, and sulfites are the examples [12]. For this reason, the application of bio-preservatives (natural antimicrobial food preservatives) can be viewed as a safe and promissory alternative to maintaining the quality of food in contrast to other preservation approaches, such as thermal and chemical methods [12]. Lately, plant polyphenols have gained pivotal attention as they exhibit positive effects on well-being by reducing the oxidative processes and curbing the growth of diverse pathogens, such as bacteria, fungi and viruses [13]. Additionally, they have also been stated to stimulate the growth of both beneficial and commensal microbes [14].

Furthermore, polyphenols’ protective effect has been well-documented and discussed, particularly with concern to how they are extracted and how to exert their biological effects in tissues (target), aspects related to metabolism and intestinal absorption [15]. In the digestive system of humans, polyphenols are metabolized via intestinal microbiota or hepatic/intestine cell. An increasing amount of evidence has discussed polyphenols’ bioavailability, metabolism, and mechanism of action while providing evidence on the beneficial effects in both animals and human models [15]. Moreover, these polyphenolic metabolites show interindividual variability in urine and plasma following ingestion, mostly correlated with the genetic polymorphisms and microbiota composition in the human gut and further linked to definite health effects [15]. Therefore, the current review intends to summarize the existing knowledge about fruit polyphenols’ antibacterial potential against foodborne pathogens. Moreover, the use of these fruit extracts in synthesizing functionalized nanoparticles will also be discussed. This knowledge is of extreme importance in the direction of developing health-promoting functional foods as well as natural antimicrobial food preservatives.

## 2. Types of Fruits and Classification of Fruit Polyphenols

Basedon the number of flowers and ovaries, fruits are separated into three categories, i.e., simple, aggregate, and composite fruits as shown in Table 2 [16].

Polyphenols can be categorized into four core classes, i.e., flavonoids, stilbenoids, tannins, and phenolic acids (Table 3).

Out of all polyphenols, flavonoids are predominantly found in fruits. According to the general structure, the flavonoid backbone comprises two phenyl rings and an oxygenated heterocyclic ring with a 15-carbon skeleton (C6–C3–C6 backbone). The most widely known flavonoids are anthocyanidins, chalcones, flavan-3-ols, flavanones, flavones, flavanonols, flavonols, and isoflavones (Table 3). The highly common flavonoids are flavones and flavonols, abundantly found in the plant kingdom, except in fungi and algae [33]. The major flavonoids involve monomeric flavan-3-ols (catechins) and their derivatives (epicatechin, gallocatechin). The aromatic ketone 1,3-diphenyl-1-propen-3-one backbone is usually found in chalcones. Chalcones are found in vegetables and fruits in the form of monomers, Diels–Alder adducts, dimers, oligomers, and in the form of various conjugates. Anthocyanidins polyphenols are claimed to impart blue, purple, and red pigments to fruits and the petals of the flower. Around >500 anthocyanins are known todate, varying in terms of the patterns of methoxylation or hydroxylation of the β-ring and glycosylation of diverse sugar units [34]. In addition, colour imparting by anthocyanins is found to be pH-dependent, with an orange or red colour meaning a pH of < or 3.0, bluish-red (pH 6–7), and blue (pH 7.5 or >) [35].

Stilbenoids are stilbene derivatives, also known as 1,2-diphenylethene polyphenols having a 14-carbon skeleton (C6–C2–C6 backbone) (Table 3). Stilbenoids can have monomeric, polymeric, or oligomeric structures. Owing to diverse biological activities, such as antibacterial, anticancer, antioxidant, estrogenic, and *trans-*resveratrol effect, these polyphenols have gained considerable attention from various researchers [36,37].

Phenolic acids are derived from benzoic acid (C1–C6 backbone) or cinnamic acid (C3–C6 backbone) (Table 3). In fruits, hydroxybenzoic acids (in freeform) are primarily found in lesser concentrations. Usually, phenolic acids are found in bound form, hydrolyzed by enzymatic action or alkaline media. Another phenylpropanoid derivative, i.e., hydroxycinnamic acid, also found in bound forms (esters of quinic acid or glycosylated derivatives) in all parts of fruits [38]. Tannic acid (also known as tannins) are water-soluble polyphenols that are found in diverse fruits, such as apples, blackberries, bananas, dates, cranberries, hawthorns, grapes, pears, peaches, plums, persimmons, strawberries, and raspberries (Table 3) [39]. The antimicrobial potential of these tannic acids is often associated with the presence of an ester linkage between polyols and gallic acid, which get hydrolyzed after ripening. Hence, tannins are claimed to play an essential role as a natural defense mechanism against microbial pathogens.

## 3. Extraction of Polyphenols from Fruits

There are various ways to extract phenolics from fruit samples either dried, fresh, or frozen. Prior to extraction, the samples must pass through multiple unit operations such as milling, grinding, drying, and homogenization [40].

### 3.1. Conventional Methods

Despite a few drawbacks, liquid and solid–liquid extraction fluids are the most commonlyutilized extraction methods. For a long time, the conventional techniques have been generally acknowledged, predominantly in terms of convenience, productivity, greater relevance, and acceptability [41,42]. These methods include the utilization of solvents such as alcohols (methanol, ethanol), diethyl ether, ethyl acetate, and acetone blended in with various extents of water. The residues of these solvents remain in the resultant products and pose a risky impact on human well-being. The removal of those residues from the samples requires tedious purification steps, ultimately impacting the processing cost. Furthermore, phenolic acids such as benzoic and cinnamic acids can be separated using combinations of alcohol–water or acetone–water as pure organic solvents cannot separate them. Moreover, highly non-polar compounds (waxes, oils, sterols, chlorophyll) may be extracted by utilizing less polar solvents such as hexane, benzene, dichloromethane, and chloroform [41].

The solvent attributes greatly influence the rate and yield of polyphenols extracted from the samples. Studies reported that methanol and aqueous acetone are more proficient in extracting polyphenols with lower atomic weight and higher molecular weight flavanols [43,44,45,46]. High processing temperatures and long processing times result in the degradation and undesirable oxidation of many phenolic compounds in the extracts. Typically, extraction is carried out at 20–50 °C and temperatures above 70 °C led to a hasty degradation of anthocyanin. Due to the high demand for organic solvents, less efficient conventional extraction methods such as maceration and soxhlet are used. In general, various factors (acidic and alkaline hydrolysis, pH of the sample, pH, and polarity of eluents) influence the phenolic extracts’ stability. Hence, a pH of 4–5 was related to improved stability of catechins and their isomers associated with acidic and alkaline conditions [47].

### 3.2. Modern Extraction Techniques

There is a dire need to advance development procedures and use extraction strategies such as supercritical fluid extraction, microwave-assisted extraction, ultrasound-assisted extraction, ultrasound–microwave-assisted extraction, and subcritical water extraction [48] as there are many issues exposed to high temperatures and long handling times in case of conventional methods. These techniques are simple, have shorter extraction times, and reduce the consumption of organic solvents. Since the use of mild conditions eludes oxidation and degrades the labile mixtures, some researchershave investigated the use of supercritical fluid technologies for the selective isolation of antioxidants from natural materials [49,50]. During this process, the products with higher quality and healthier nutrients are obtained. Recently, due to legal constraints, the removal of solvent residues has been limited the utilization of conventional organic solvents in the fields of the food and drug industries. Nowadays, alternative production technologies have replaced traditional production technologies with minimal environmental impact and low toxic waste yield.

Several research papers have examined the use of supercritical extraction for determining and quantifying of phenolic compounds from different materials and their use as additives. The compound should have an attribute of high solubility in the supercritical solvent for high economy in supercritical fluid extraction process [51]. In this regard, the mass transfer of target compounds and the resulting yield must be considered [52]. Further, to obtain the best ratio between the yield, solvent amount, and extraction time, the pressure drop effect must be evaluated and considered. Proper sample handling procedures have to be carried out to isolate bioactive polyphenols from plant matter before extraction.

Supercritical fluid solvents act as an intermediate between liquids and gases by escalating the fluid’s density and increasing the solubility of the compound. The viscosity, which is equivalent to gas viscosity, allows for improved transport characteristics. The key benefit of supercritical fluids is the prospect of drastically modifying the solvent properties near their critical point. Solvent selectivity also represents a significant aspect of the solvent and varies significantly with pressure and temperature. It is also observed that there is low solvent selectivity in a system with high solubility strength and it is possible to improve the later by adding a co-solvent [53].

Supercritical CO_2_ is the solvent of choice for extraction processes due to ease of penetration within fruit materials and high solvent power. In addition to CO_2,_ there are numerous alternatives to supercritical fluids used for extraction purposes. Cosolvents and supercritical fluids such as propane, argon, and SF6 are executed in processing compounds of low polarity and low molecular weight. Due to the high critical temperature and pressure, high energy consumption, and the corrosive nature of H_2_O in the supercritical state, limited water is used in practical applications [54]. In extracting phenolic compounds, subcritical water extraction has become an increasingly common alternative technology. In certain situations, such as in the use of cosolvents for extracting more polar compounds from aromatic plants, water is often applied to the system. A highly useful feature of both pressure and temperature is the dielectric constant of water. In the domain of the critical point, a slight change of pressure can easily fine-tune the dielectric constant and polarity. The polarity decreases under subcritical conditions because of the breakdown of intermolecular hydrogen bonds. For example, at room temperature, water has high polarity and a dielectric constant near 80. The dielectric constant decreases dramatically by increasing the pressure at the temperature of 250 °C and becomes similar to that of ethanol [55,56]. This implies that the inorganic and organic components can be extracted using the same solvent. The main advantage of supercritical extraction over conventional methods is its simplicity, high quality extract, low extraction time and environmental friendliness due to water being used as the solvent.

## 4. Antibacterial Mechanism of Fruit Polyphenols

**Figure 1:**
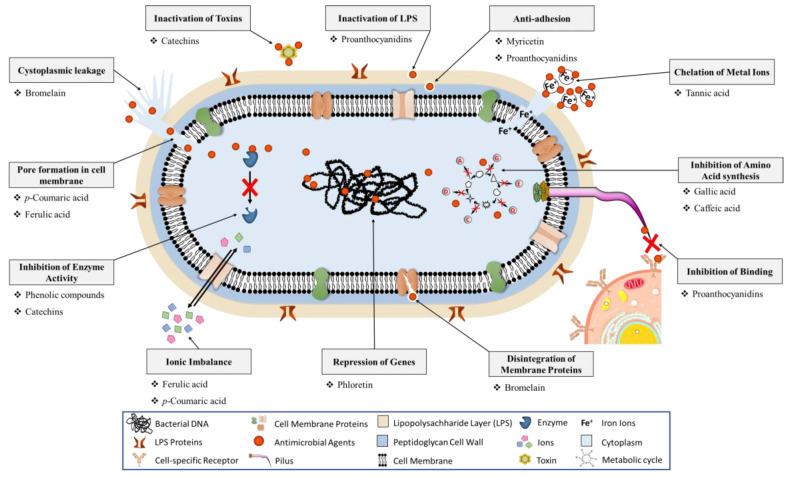
Antibacterial mechanism of fruit polyphenols with their target sites.

**Figure 2:**
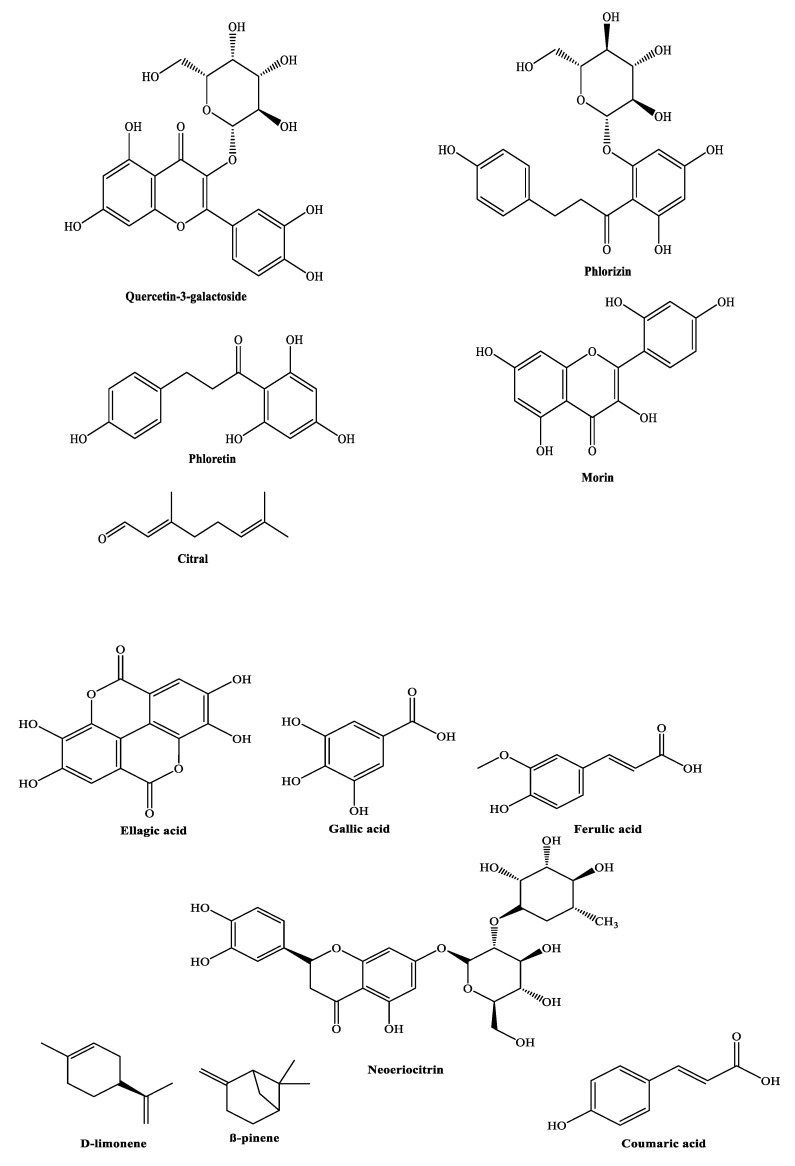

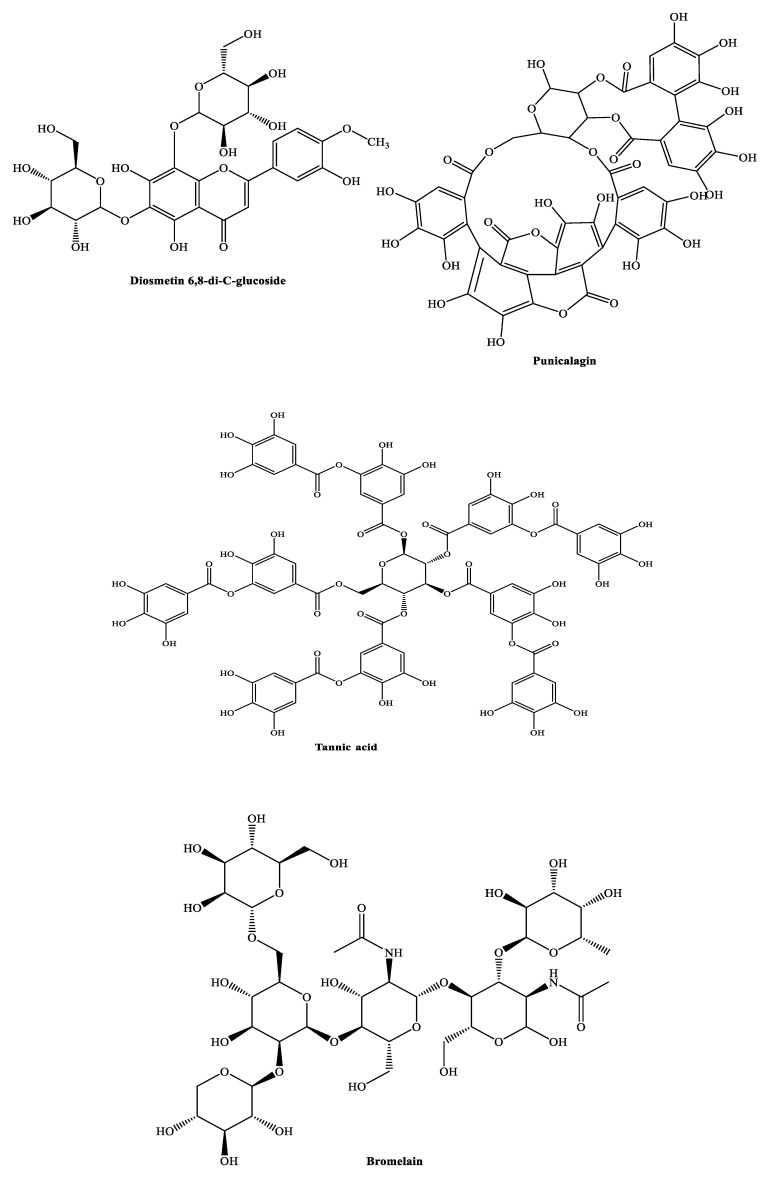
Various types of antibacterial polyphenols present in fruits.

The interaction of fruit polyphenols with different nonspecific forces, such as hydrogen bonding, covalent bond formation, and hydrophobic and lipophilic interactions, has been associated with adhesins, cell envelope transport proteins, enzymes, and microbial membranes, as depicted in Figure 1 [57,58]. Indeed, polyphenols are claimed to exhibit antibacterial activity owing to their ability to chelate iron, which is essential to all bacteria for their survival [59]. Few fruit polyphenols showing antibacterial activity are illustrated in Figure 2.

### 4.1. Interaction with Cell Wall and Cell Membrane

The cell walls of both Gram-positive and Gram-negative bacteria are different. For instance, in Gram-negative bacteria, the cell wall comprises an outer membrane (OM) and a thin layer of peptidoglycan. The OM is further made up of protein and a phospholipid bilayer, and the outer leaflet of the membrane encompasses lipopolysaccharides (LPS). In contrast, Gram-positive bacteria cell wall lacks OM and contains lipoteichoic acid and a thick layer of peptidoglycan [60]. In addition, Gram-positive and Gram-negative cell walls have been stated to play an imperative role in osmotic protection. Therefore, it is stated that damaging the bacterial cell wall reduces their tolerance to low osmotic pressure and high ionic strength. The literature has shown that flavanols, flavonoids, and flavones exhibit very effective antimicrobial effects against numerous pathogenic microbes [61,62,63].

Zhao et al. [63] reported that epigallocatechin gallate (EGCG) directly binds to the peptidoglycan of *Staphylococcus aureus,* altering the cell integrity and reducing cell tolerance to low osmotic pressure and high ionic strength. Yoda et al. [64] also conducted a study to address the antibacterial potential of EGCG against different *Staphylococcus* strains and Gram-negative rods. Different susceptibilities to EGCG were stated, attributed to differences in EGCG affinity to different cell wall components. In addition, extracts of black currant, blueberry, cranberry, and cloudberry have been stated to have the potential to release LPS from *Salmonella enterica* serovar Infantis VTT E-97738 and *Salmonella enterica* serovar Typhimurium VTT E-981151, similar to EDTA [65]. Nohynek and his colleagues [65] reported that the phenolic extract of cloudberry and raspberry could disintegrate the OM of *Salmonella* strains via chelating divalent cations.

Moreover, they stated that ellagic acid from cranberry and ellagitannin from cloudberry and raspberry were responsible for showing antimicrobial activity. Delehanty et al. [66] reported that proanthocyanidins obtained from cranberries bind to bacterial LPS and neutralize its charge, explaining the inhibition of the binding of LPS to the surface of mammalian cells. Johnson et al. [67] conducted a study using immobilized proanthocyanidins obtained from cranberry juice, grape juice, and whole cranberries and stated a good ability to arrest the LPS of the bacterial cell. The explanation for this activity was attributed to phenolic compounds’ ability to bind to lipid components of LPS. *Fimbriae* and *pili*, an important bacteria component, play an effective role during the adhesion to the host tissue. The lectin-like mechanism is found to be responsible for the binding of these protein filaments to complementary carbohydrates receptors of the host cell tissue. It is known that polyphenols, specifically proanthocyanidins, can obstruct the pili binding to cell-specific receptors [68]. The competitive inhibition mechanism of this polyphenol is explained by considering proanthocyanidins as receptor analogues.

It has been reported that fruit polyphenols interact with phospholipids or proteins of the lipid bilayer. These polyphenols interact with Gram-positive and Gram-negative bacteria membrane and disrupt the lipid bilayer, ultimately increasing membrane permeability, affecting its fluidity, altering the ion transport process, and inhibiting respiration [69]. Wu et al. [70] conducted a study to assess the antibacterial potential of five flavonoids (baicalein, chrysin, kaempferol, luteolin, quercetin), four isoflavonoids (puerarin, daidzein, ononin, genistin), and two polymethoxyflavones (5,6,7,4′-tetramethoxyflavone, tangeritin) against *Escherichia coli.* The results obtained revealed that the antibacterial activity decreased in the following order: flavonoids *>*polymethoxyflavones*>*isoflavonoids. Borges et al. [71] conducted a study to assess ferulic and gallic acids’ abilities to induce irreversible changes in the membrane properties of *Listeria monocytogenes, S. aureus*, and *E. coli*. The study results revealed that the interaction of ferulic and gallic acids with the cell membrane causes a decrease in surface charge (negative charge), pore formation, and caused leaking of intracellular constituents and hydrophobicity changes. Another useful bioactive molecule found in fruits such as apple, pear, and kinnow is *p*-coumaric acid, which shows antibacterial activity against numerous bacteria, such as*Bacillus cereus*, *Bacillus subtilis*, *Shigella dysenteriae*, and *Salmonella typhimurium* [19,72]. The antimicrobial potential of *p*-coumaric acid depends on pH, as the minor decrease in pH elevates these molecules’ antimicrobial activity against different microbial strains. Therefore, the mechanism of action of *p*-coumaric acid involves changes in membrane permeability and pore formation.

### 4.2. Interaction with Enzymes

Recent studies conducted using polyphenols have suggested that these bioactive molecules can inhibit cyclic di-AMP synthase activity that catalyzes cyclic-di-AMP biosynthesis and is involved in various cellular processes [73,74,75,76]. Xiao et al. [77] investigated 19 flavonoid targets in *E. coli* with comparative genomics and molecular modelling. The result recognized the listed enzymes dihydroorotate dehydrogenase, DNA gyrase subunit, dihydrofolate reductase, and fumarate reductase flavoprotein NADH-dependent enoyl-ACP reductase.

### 4.3. Interaction with Protein

Phloretin, an apple flavonoid, was also stated to control biofilm formation in *E. coli* O157:H7 via a mechanism that suggests the curbing of curli genes, i.e., csgA and csgB, that are involved in the production of fimbriae [78]. Bromelain (a protein-degrading enzyme) is also found in rich amounts in fresh pineapples and is often used for tenderizing meat [79]. Different studies have suggested that bromelain weakens the outer membrane by disintegrating the surface membrane protein in Gram-negative bacteria, leading to leaking, swelling, and damaging cells [80].

## 5. In Vitro Antibacterial Activity of Fruit-Polyphenols-Rich Extracts

Numerous in vitro studies have illustrated the antimicrobial potential of natural phenolic compounds against foodborne pathogens. The antibacterial activity of different fruits extract against several foodborne pathogens is listed in Table 4.

Vallejo et al. [83] conducted an experiment to assess the antibacterial potential of low-molecular-weight phenolic fractions (LMPFs) of *Albion* (LMPF-A) and *Camarosa* (LMPF-C) strawberry juice against *Listeria monocytogenes* and *S. typhimurium.* The bioactive molecule quercetin was found to play an imperative role in both phenolic fractions. The potential antibacterial activity of these phenolic extracts was associated with releasing potassium and phosphate ions, the disintegration of the cell membrane and the inhibition of NADH oxidase. Silvan et al. [23] also conducted a study to assess the antibacterial potential of plum extracts powder (PEP) against five foodborne bacteria (*C. jejuni*, *E. coli*, *L. monocytogenes*, *S. aureus*, and *S. typhimurium*). As the main results, the freeze-dried (FD) extract revealed a highly active bactericidal effect, and quercetin-3-O-galactoside (hyperoside) was found to be present in high quantities, thus concluding that hyperoside could be responsible for the antimicrobial effect, given that a significantly higher concentration of this compound was found in the powder obtained via freeze-drying.

In contrast, other phenolic compounds were found in lower concentrations. Moreover, the methanol peel extract of seven different pomegranate cultivars (Arakta, Ruby, Bhagwa, Herskawitz, Ganesh, Wonderful, and Molla de Elche) was reported to show broad-spectrum antimicrobial activity against both Gram-negative (*E. coli*) and Gram-positive (*S. aureus* and *B. subtilis*) bacteria [88]. All cultivars were found to contain catechin, ellagic acid, epicatechin, and gallic acid, of which ellagic acid was found to be in higher amounts, accounting for 50% of the total phenolic compounds in each cultivar.

Loon et al. [90] reported acetone extract of pineapple pulp minimum inhibitory concentration (MIC) in the range of 1.56–0.78% against *S. aureus*. The study results revealed that bromelain, flavonoid, and vitamin C were the main bioactive constituents present in pineapple extract exhibiting antibacterial activity. In addition, Singh et al. [92] conducted a study using the polyphenol extract of fruit jamun/jambolana to assess the antimicrobial potential against *E. coli, S. aureus*, and methicillin-resistant *S. aureus* (MRSA) and found MIC and zone of inhibitions of 0.5–2.5 mg/mL and 14.3–23.0 mm, respectively. Xu et al. [96] found that the seed polyphenol and skin extract of muscadine grape displayed effective antimicrobial activity against *S. aureus* and little-to-no antibacterial activity against *E. coli* O157:H7, *S. sonnei* ATCC 25931*,* and *S. typhimurium,* thus concluding that the antibacterial activity of phenolic compounds of the muscadine grape was explicitly not dependent on the concentration oron the specific phenolic compound. Similarly, a study conducted to assess the antibacterial potential of four blueberry cultivars (Bluecrop, Duke, Darrow, and Elliot) showed a dose-dependent inhibition of growth of *S*. *enterica* serovar Enteritidis and*L. monocytogenes*, where chlorogenic acid, ellagic acid, quercetin, and quercetin-3-galactoside were revealed to be the active phenolic compounds responsible for the antibacterial activity of blueberry extracts [24].

## 6. In Vitro Antibacterial Activity of Polyphenol-Functionalized Nanoparticles (NPs)

**Figure 3:**
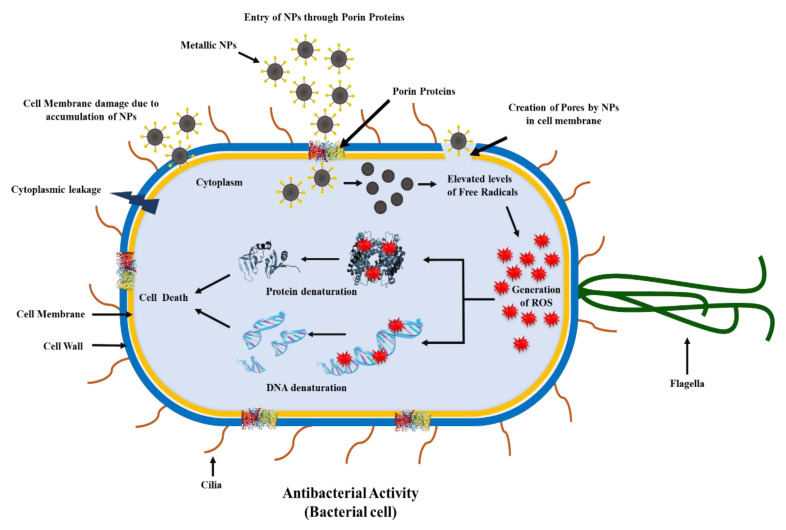
Antibacterial mechanism of fruit-polyphenol-functionalized nanoparticle with their target sites [119].

The extracts of fruits such as blueberries, blackberries, *Cornus mas* L., *Citrullus lanatus*, grape, *Terminalia arjuna*, and *Punica granatum* L. are comprised of polyphenols [103] and have been reported to contain reducing agents in high amounts. The fruit-polyphenol-functionalized NPs have an additional advantage as compared to the NPs synthesized by the biological method. NP synthesis by biological method uses microbes of pure strains and must be maintained in an aseptic environment. However, their separation from microbial broth culture during downstream processing is difficult [104]. The various types of polyphenol-functionalized NPs using fruit extracts and their antibacterial activity has been shown in Table 5. The antibacterial mechanisms of NPs are represented in Figure 3.

The use of *Ziziphus spina-christi* (L.) extract in the derivatization of Cu_2_ONPs has been reported to demonstrate antimicrobial activity against *S. aureus* compared to *E. coli* [105]. In addition, Cu_2_ONPs from fruit extract of *Capparis spinosa*, exhibited antimicrobial activity against *Bacillus cereus* and *S. aureus* in contrast to *E. coli* [106]. The bactericidal effect of AgNPs obtained from orange juice was observed against various concentrations, and the concentrations of 5 and 10 μg/mL were reported to not be effective in terms of a bactericidal effect. In contrast, a concentration of 20 μg/mL, 30 μg/mL, or 40 μg/mL was found to be bactericidal for *B. subtilis, Shigella, E. coli*, and *S. aureus*. Moreover, *Citrus maximas* extracts used for ZnONPs exhibited considerable antimicrobial activity against *S. aureus* and minimal antimicrobial activity towards *E. coli* [118]

## 7. Safety Issues and Current Challenges

Some studies have been conducted primarily focusing on the safety and toxic features of polyphenols consumption. For example, the direct ingestion of moderate doses of resveratrol is considered cardioprotective and safe [120]. Similarly, the consumption of resveratrol supplements has also not shown any severe or detrimental effects confirmed by animal study, suggesting it to be safe for use and indicating beneficial effects [121]. On the other hand, grape seed extract was found to be safe following a repeated dose administered in healthy rats and exerted effective anti-inflammatory and antioxidant activities [122]. On the contrary, the intraperitoneal administration of EGCG (high dosage) in diabetic mice showed cardiotoxicity [123].

Moreover, the limited efficacy of polyphenols as a result of their low bioavailability ratios has been increasingly addressed. In this way, nanotechnology has been increasingly used as a way to overcome such constraints. Specifically, encapsulation is an innovative pharmaceutical formulation that allows the target molecule to reach the targeted site and avoid their loss due to metabolism or even the occurrence of adverse effects. Still, poor regulatory constrictions of non-pharmaceutical formulations and commercially available polyphenol supplements are a reason of concern for their safe usage. There are some concerns regarding polyphenol fortification and supplementation. For example, their consumption may replace the intake of healthy whole foods, such as fruits and vegetables.

There is a lack of synergistic effects and health benefits in polyphenols supplementation and diet fortification in comparison of diets that are naturally rich in polyphenols [124]. The health benefits include the consumption of a high-fibre diet, potentially interacting nutrients and non-nutrients, and satiation. To understand the complex interactions underlying the functional benefits with the consumption of whole foods containing polyphenols is a great challenge in the area of polyphenol research [125]. In comparison to epidemiological studies, the consumption of isolated polyphenolic compounds alone may not produce the same effects. Instead, nutrient-dense, fortified foods can also be more energy-dense, which further balances the anti-obesogenic effect of polyphenols and leads to weight gain [126]. Lastly, the cost involved in extracting natural phenolic compounds is affected by the degree of purification, with the extract quality being improved by removing toxic organic constituents [127].

## 8. Conclusions

Numerous fruit polyphenols have been found to exhibit promising antibacterial activity by curbing the growth of biofilm-forming pathogens associated with food. The examination of this potential is chiefly crucial as bacterial biofilms formed on food and surfaces that comes in contact with food results in microbes resistant to conventional disinfectants. Additionally, the inhibitory potential of fruit polyphenols against enterotoxin activity and production makes them effective bioactive molecules to prevent food illness. Moreover, fruit polyphenols have been stated to regulate toxin production and biofilm formation effectively. Hence, the discovery and analysis of substances able to suppress the growth of pathogenic microbes have occupied a prominent place in current research. In short, more emphasis should be given to exploring these fruit polyphenols so that they can be used as food preservatives in food industries. Furthermore, the utilization of fruit polyphenols in synthesizing nanoparticles has the additional advantage of being economical, cost-effective, eco-friendly, and energy-efficient. Therefore, the utilization of fruit polyphenols in synthesizing nanoparticles would bring a boom to this field in the coming years. Even though extensive literature is available related to nanoparticle synthesis using fruit extracts, the approach of using fruit polyphenols as reducing agents for nanoparticle synthesis still remains overlooked. As a result, the exploration of fruit polyphenols capable of synthesizing nanoparticles has unveiled the new avenues and has become anemerging field.

## Figures and Tables

**Table 1:** Disease outbreaks due to foodborne bacteria.

Country	Year	Source	Pathogen	Disease	Confirmed Cases	Ref.
Australia	2001–2007	Chicken	*Campylobacter jejuni*	*Campylobacteriosis*	16,000	[4]
Brazil	2013	Cooked salads	*Staphylococcus aureus*	Food illness	472	[5]
Canada	2015–2019	Frozen raw chicken products	*Salmonella enterica* serovar Enteritidis	*Salmonellosis*	584	[3]
France	2007–2014	Starchy foods and vegetables	*Bacillus cereus*	Diarrhoea	911	[6]
Germany	2001–2002	Chocolate	*Salmonella (S.)* Oranienburg	*Salmonellosis*	439	[7]
Greece	2019	Minced beef	*Clostridium perfringens*	Gastroenteritis	71	[8]
India	2016	Unrefrigerated raw sliced tomatoes	*Shigella sonnei*	Gastroenteritis	34	[9]
Japan	1996	White radish sprouts	*Escherichia coli* O157:H7	Diarrhoea	7000	[10]
Saudi Arabia	2009	Local sweet	*Salmonella enteric* serovar Enteritidis	*Salmonellosis*	200	[11]
United Kingdom	2017	Chicken liver dishes	*Campylobacter* spp.	*Campylobacteriosis*	7	[3]
United States	2013–2014	Chicken dishes	*Salmonella* Heidelberg	*Salmonellosis*	634	[3]
Zimbabwe	2014	Stewed chicken	*Staphylococcus aureus*	Food illness	53	[3]

**Table 2:** Categorization of fruits.

Category	Description	Example
**Simple**	Simple fruits are those developed into fruit from the mature ovary of the flower	
*Drupes*	Also known as stone fruits, such fruits comprise hard seed within fruits	Cherry, peach, plum
*Berries*	Juicy and single seed fruits, with seeds being found at the center	Banana, blueberries, grapes, pomegranate
*Pomes*	Fruits that blossom in the trees	Papaya, apple
*Hesperidium* and *Pepos*	Fruits often proclaimed to be slightly similar to berries and comprises fruits	Citrus fruits
**Aggregate**	These fruits develop by merging numerous matured ovaries, which were previously a single flower	Strawberry
**Composite**	These fruits are also stated as multiple fruits as they develop from complete inflorescences	
*Sorosis*	Fruits developed from spadix, spikes, or catkin inflorescence	Pineapple, jackfruits, mulberry
*Syconus*	Fruits developed from hypanthodium inflorescence	Fig

**Table 3:** Polyphenols: classes, subclasses, and fruit sources.

Class Name, Subclass Name	Examples	Sources	References
**Flavonoids**			
*Flavones*	Luteolin, Apigenin	Fig, Grape	[17,18]
*Flavanones*	Hesperidin, Naringenin	Kinnow, Grape, Orange, Citron	[18,19,20,21]
*Flavonols*	Quercetin, Quercetin-3-O-galactoside, Kaempferol, Myricetin, Morin	Kinnow, Mulberry, Fig, Grape, Plum, Blueberry, Cherry, Black Plum, Apple, Pomegranate, Guava, Strawberry	[17,18,19,22,23,24,25,26,27,28,29,30]
*Flavan-3-ols*	***Monomers:*** (+)-Catechin, (−)-Epicatechin, (−)-Epigallocatechin, (−)-Epicatechin-3-gallate, (−)-Epigallocatechin-3-gallate	Banana, Kinnow, Mulberry, Fig, Grape, Pomegranate	[17,18,19,22,28,31]
*Isoflavones*	Genistein, Daidzein, Dihydrodaidzein, Equol	Grape	[18]
*Anthocyanidins*	Cyanidin, Pelargonidin, Peonidin, Delphinidin, Petunidin, Malvidin, Cyanidin-3-glucoside, Cyanidin-3-rutinoside, Pelargonidin-3-glucoside	Mulberry, Fig, Grape, Orange, Plum, Cherry, Black Plum, Pomegranate, Strawberry	[17,18,19,20,21,22,23,25,26,28,30]
*Dihydrochalcone*	Phloridzin, Phloretin	Apple	[27]
**Stilbenoids**			
*Stilbenoids*	*trans-*Resveratrol, *trans-*Piceid	Grape	[18]
**Phenolic acids**			
*Benzoic acids*	***Monomers:** p*-Hydroxybenzoic acid, Gallic acid, Protocatechuic acid(3,4), Cinnamic acid, ellagic acid	Banana, Kinnow, Fig, Grape, Blueberry, Black Plum, Apple, Pomegranate, Guava	[17,18,19,24,26,27,28,29,31]
*Hydroxycinnamic acids*	Caffeic acid, *p*-Coumaric acid, Ferulic acid, Synaptic acid ***Chlorogenic acids:** *Chlorogenic acid	Banana, Kinnow, Mulberry, Fig, Grape, Plum, Black Plum, Apple, Pomegranate, Strawberry	[17,18,19,22,23,26,27,28,30,31]
*Quinic acids*	Neochlorogenic acid, 3-feruloylquinic acid, 3-O-p-Coumaroylquinic acid	Plum	[23]
**Tannin**			
*Tannic acid*	***Monomers:*** Tannic acid	Pomegranate, Star Apple	[28,32]

**Table 4:** Antibacterial activity of some fruits against foodborne pathogens.

Scientific Name	Common Name	Extract	Bacteria	References
*Psidium guajava* L.	Guava	Aqueous; Methanol	*S. aureus* ATCC 25923*, E. coli* ATCC 25922*, B. cereus* BTCC 19, *S. sonnei* BTCC and *S. typhi* BTCC 197; *S. aureus* ATCC 29213	[81,82]
*Fragaria* x *ananassa*	Strawberry	Aqueous	*L. monocytogenes* and *S.* *typhimurium*	[83]
*Carica papaya* L.	Papaya	Methanol; Ethanol	*E. coli* ATCC 25923, *S. typhi* ATCC 14028, *B. cereus* ATCC 11778, *B. subtilis* ATCC 11774; *S. aureus, S. dysenteriae, S. typhi*, *E. coli*	[84,85]
*Prunus domestica* L.	Plum	Aqueous	*C. jejuni*NCTC11168*,E. coli* ATCC^®^25922*,* *S. aureus* ATCC^®^25923*,* *L. monocytogenes* CECT935*,* and *S. enterica* subsp. enterica serovar *typhimurium* ATCC^®^ 14028	[23]
*Punica granatum* L.	Pomegranate	Ethanol; Methanol	*S. aureus*; *E. Coli* ATCC 11775, *B. Subtilis* ATCC 6051, *S. aureus* ATCC 12600	[86,87,88]
*Ananas comosus* L.	Pineapple	Ethanol, Aqueous; Acetone	*E. coli*, *B. cereus, S. aureus*	[89,90]
*Syzygium cumini* L.	Jamun	Aqueous; Ethanol	*S. typhimurium*, *S. flexneri*, *S. aureus*, ETEC (Enterotoxigenic *E.coli*); *S. aureus*, *E. coli*	[91,92]
*Citrus* x *aurantium*	Sour Orange	Aqueous	*L. monocytogenes* and *S.* *typhimurium*	[93]
*Citrus* x *sinensis*	Sweet Orange	Ethanol; Methanol	*S. aureus*, *E. coli*, *S.typhimurium*; *S. aureus*, *S. flexineri, B. subtilis, E. coli*	[94,95]
*Vitis rotundifolia* Michx.	-	Methanol	*S. aureus* strains ATCC 35548, *S. typhimurium*, *S. sonnei* ATCC 25931, *E. coli* O157:H7	[96]
*Vitis vinifera* L.	Grape	Acetone	*L. monocytogenes* ATCC 7644, *S. aureus* ATCC 29213	[97]
*Vaccinium corymbosum* L.	Blueberry	Ethanol	*Vibrio parahaemolyticus;L. monocytogenes* and *S.* *enteritica* serovar Enteritidis	[24,98]
*Ficus carica* L.	Fig	Methanol	*S. aureus* ATCC 25923, *E. Coli* ATCC 25922	[99]
*Musa paradisiaca* cv. Puttabale	Banana	Ethanol: Hexane, Acetone, Ethanol, Water	*B. subtilis* NCIM2063, *S. aureus* NCIM2079, *S. typhi* NCIM 2501, *S. paratyphi* MTCC735; *B. cereus* DPMB 1, *S. aureus* ATCC 6538, Rosenbach, *S. enterica* subsp. enteric ATCC 13076; *S. sonnei* LMG 10473	[100,101]
*Malus domestica cv. Gala*	Apple	ND	*S. aureus*, *L. monocytogenes*	[102]

ND: not defined.

**Table 5:** Antibacterial activity of functionalized nanoparticles against foodborne pathogens.

Scientific Name	Common Name	Biological Extract	Types of NPs Synthesized	Reaction Temperature/Time	Morphology	Size	Bacteria	References
*Ziziphus spina- christi* (L.) Willd	Christ’s thorn jujube	Pulp	Copper oxide	80 °C/NS	Sphere	5–20 nm	*E. coli* and *S. aureus*	[105]
*Capparis spinosa*	Caperberry	Whole fruit	Copper oxide	80 °C/24 h	Sphere	17–41 nm	*E. coli*, *S. aureus* and *B. cereus*	[106]
*Citrus medica* Linn.	Citron	Juice	Copper oxide	60–100 °C/NS	NS	10–60 nm	*E. coli* and *S. typhimurium*	[107]
*Fragaria* x *ananassa*	Strawberry	Whole fruit	Copper oxide	RT/1 h	Sphere	10–30 nm	*S. aureus*, *S. typhimurium*, *B. subtilis* and *E. coli* O157:H7	[108]
*Punica granatum* L.	Pomegranate	Juice	Silver	65 °C/1 min	Cubic	23 nm	*E. coli* and *S. aureus*	[109]
*Carica papaya* L.	Papaya	Juice	Silver	NS	Sphere	75.68 nm	*E. coli* and *S. aureus*	[110]
*Vitis vinifera and Lycopersicon esculentum* Mill.	Grape and Tomato	Juice	Silver	RT/NS	Cubic	10 and 30 nm	*S. aureus*, *B. subtilis* and *S. typhimurium*	[111]
*Hylocereus undatus* (Haworth)	Dragon fruit	Peel	Silver	RT/24 h	Sphere	25–26 nm	*E. coli* and *S. aureus*	[112]
*Citrus limetta* Risso	Sweet lime	Juice	Silver	RT/24 h	Quasi-sphere	5–35 nm	*E. coli*, *S. aureus* and *Yersinia enterocolitica* subsp. *enterocolitica*	[113]
*Punica granatum* L.	Pomegranate	Juice	Silver	RT/4h	Sphere	30–40 nm	*B. subtilis*	[114]
*Citrus* x *sinensis*	Orange	Juice	Silver	37 °C/2 h	NS	NS	*S. aureus*, *B. subtilis*, *E. coli* and *Shigella*	[115]
*Prunus armeniaca* L.	Apricot	Peel	Silver	NS	Rod	50 nm	*S. aureus*, *B. subtilis* and *E. coli*	[116]
*Ananas comosus* L.	Pineapple	Juice	Zinc oxide	240 °C/5 min	NS	30–57 nm	*E. coli*	[117]
*Citrus maxima* Merr.	Pomelo	Juice	Zinc oxide	400 °C/5–10 min	Agglomerated	10–20 nm	*E. coli* and *S. aureus*	[118]

RT—room temperature; NS-not specified.

## Data Availability

Not applicable.
